# Pedigree analysis of pre-breeding efforts in *Trifolium* spp. germplasm in New Zealand

**DOI:** 10.1186/s12863-020-00912-9

**Published:** 2020-09-14

**Authors:** L. M. Egan, R. W. Hofmann, P. Seguin, K. Ghamkhar, V. Hoyos-Villegas

**Affiliations:** 1 AgResearch Lincoln Research Centre, Christchurch, PB 4749 New Zealand; 2 Faculty of Agriculture and Life Sciences, Lincoln University, Lincoln, New Zealand; 3 Faculty of Agricultural and Environmental Sciences, Department of Plant Science, McGill University, Montreal, Canada; 4 AgResearch Grassslands Research Centre, Palmerston North, PB 11008 New Zealand

**Keywords:** Pedigree, *Trifolium*, Prebreeding, Clover, Relatedness, Germplasm, Ancestors

## Abstract

**Background:**

Prebreeding in plants is the activity designed to identify useful characteristics from wild germplasm and its integration in breeding programs. Prebreeding aims to introduce new variation into the populations of a species of interest. Pedigree analysis is a valuable tool for evaluation of variation in genebanks where pedigree maps are used to visualize and describe population structure and variation within these populations. Margot Forde Germplasm Centre (MFGC) is New Zealand’s national forage genebank and holds a collection of ~ 75 species of the genus *Trifolium*, of which only a dozen have been taken through prebreeding programs. The main objective of this study was to construct pedigree maps and analyse patterns of relatedness for seven minor *Trifolium* species accessions contained at the MFGC. These species are *Trifolium ambiguum, Trifolium arvense, Trifolium dubium, Trifolium hybridum, Trifolium medium, Trifolium subterraneum* and the *Trifolium repens* x *Trifolium occidentale* interspecific hybrids. We present a history of *Trifolium* spp. prebreeding in New Zealand and inform breeders of possible alternative forage species to use.

**Results:**

Pedigree data from accessions introduced between 1950 and 2016 were used and filtered based on breeding activity. Kinship levels among *Trifolium* spp. remained below 8% and no inbreeding was found. Influential ancestors that contributed largely to populations structure were identified. The Australian cultivar ‘Monaro’ had a strong influence over the whole population of accessions in *T. ambiguum*. *T. subterraneum* and *T. repens* x *T. occidentale* had the largest number of generations (3). *T. ambiguum* and *T. medium* had the highest cumulative kinship across the decades.

**Conclusions:**

We conclude that there are high levels of diversity in the seven *Trifolium* spp. studied. However, collection and prebreeding efforts must be strengthened to maximize utilization and bring useful genetic variation.

## Background

The earliest recorded use of legumes is in the grasslands of the Mediterranean basin and today they are used in agricultural pasture systems [50]. The importance of genus *Trifolium* was recognised very early on by naturalists and herbalists. It was previously defined as a much broader genus and included two other genera, *Lotus* and *Melilotus* [32].

To date, the *Trifolium* genus of the *Leguminosae* family is made up of 250 species [8, 34, 38, 52, 60, 83, 107]. They are distributed throughout the temperate and subtropical regions of both northern and southern hemisphere, particularly in Europe, northwest and central Asia, northeast Africa, parts of sub-tropical Africa and South Africa, western North and South America, Australia and New Zealand. Approximately 25 species are of significance as feed for ruminant animals, of which 16 are economically important [34, 38, 89]. Russel and Webb [79] state that the *Trifolium* species are among the most important and valuable forage legumes in the world. Nitrogen fixation is a quality which has driven the use of *Trifolium* species in pastoral systems. However, many *Trifolium* species are underutilised in agricultural systems or their use is yet to be defined in agriculture [60].

The Margot Forde Germplasm Centre (MGFC) is New Zealand’s national germplasm centre for grasslands plant species. The role of this genebank is to collect, replenish, conserve and distribute accessions of forage species to be used for research or breeding. The collection contains over 65,000 wild accessions from more than 100 countries, comprising 2200 species from 350 genera and over 70 plant families from wild collections, foreign and domestic cultivars, breeding lines and genetic stocks. There are six *Trifolium* species that are well represented with pedigree information in the MFGC database. These are *T. arvense, T. subterraneum, T. ambiguum, T. dubium, T. hybridum* and *T. medium*. These species have been subject to breeding programmes from the 1950s, and some species have been hybridised to breed *Trifolium* interspecific hybrids (ISH). The ISH programmes have used white clover as the recurrent parent and hybridised it with a closely related species to improve targeted traits in white clover. For example, *T. occidentale* is well-adapted to dry habitats and could be a potential source of drought-tolerant genes for white clover [45].

The *T. occidentale* x *T. repens* ISH is an important breeding programme for the improvement of white clover in New Zealand. Another species, *T. arvense*, commonly known as rabbitfoot clover, is an annual clover that is native to most of Europe, excluding the Arctic zone, and western Asia. It can grow in a broad range of soil types, but prefers sandy or non-irrigated land [75]. It is used in short-lived pastoral systems and low fertility pastures, often in dry hill country environments [93]. Hancock et al. [40] used genetic modification (GM) to integrate the transcription factor, *TaMYB14*, from *T. arvense* into *T. repens* [10, 25, 27]. The GM product has the potential to decrease methane emissions and reduce bloating in livestock [40]. This is due to the increased level of proanthocyanidins which reduce the level of protein degradation in the rumen, decreasing gas and foam formation [6].

Another clover species, *T. subterraneum*, or subterranean clover, is an annual species native to the Mediterranean region, West Asia and the Atlantic coast of Western Europe. Subterranean clover is sown in over 29 million hectares worldwide and has the most contribution to livestock feed production among all annual clovers [48]. Subterranean clover has a unique characteristic where it buries its seeds so that the seed development occurs underground. This specialty enables subterranean clover to thrive in poor quality soil and drought regions, making it a viable option for dryland farmers [11, 65]. Subterranean clover is one of the most commonly grown forage crops in Australia due to its ability to withstand the extreme drought and soil types, and is a source of high quality forage [11, 65]. Since the introduction of subterranean clover into Australia, more than 40 cultivars have been bred and released [65]. Subterranean clover is a diploid (2n = 2x = 16), mostly self-pollinating species, with a genome size of 540Mpb [4, 28, 36, 42, 48].

A genetically diverse species, *T. ambiguum*, most commonly known as ‘Caucasian’ or ‘Kura’ clover, is a species native to Asia and the Caucasus (Armenia, Ukraine, Turkey and Iran) and was introduced to North America and Australasia [16, 90]. It is a rhizomatous perennial, found naturally up to high altitudes and is adapted to a wide range of environmental conditions [26, 103]. The large root and rhizome mass and persistency of Caucasian clover has made it desirable in agricultural environments exposed to extreme heat, drought and cold. However, the slow establishment rate, very specific rhizobial requirements, and inability to produce commercially viable amounts of seed has decreased the appeal of sowing it in a pasture system [16, 60]. Trials in the high country of the South Island of New Zealand have shown that, in comparison to white clover, *T. ambiguum* increases the legume content of a pasture in competition with grasses, and therefore potential nitrogen fixation in paddocks [19]. *T. ambiguum* exists in diploid (2n = 16), tetraploid (2n = 32) and hexaploid forms (2n = 48). The ploidy of the species affects traits such as flowering date and persistence but is not directly related to overall yield [16, 26]. Diploids are often the first to flower and are more persistent than tetraploids and hexaploids [26, 96].

A group of three other species, namely, *T. dubium, T. hybridum and T. medium* are less common in pastoral systems and have been predominantly used in research*.* The first species, also known as ‘Suckling clover’, is native to Europe and is a cross between *T. campestre* and *T. micranthum* [41]. It is an allotetraploid species (2n = 4x = 30) [17]. Alsike clover or *T. hybridum* is a clover that originates from continental Europe but has established in the British Isles and throughout the temperate regions of the world. It is often grown for hay or silage, highly self-sterile [99] and unlike the name suggests, is not of a hybrid origin [99]. In New Zealand, it is used often in the South Island hill country for pasture and hay. It is adaptable to a wide range of conditions and has rapid establishment [97]. The last species in this group, *T. medium*, commonly known as ‘Zig-zag clover’, is a native European species and is similar in appearance to red clover but with narrower leaflets and no white leaf markings [20]. It is a rhizomatous perennial clover with long persistence and has the ploidy of 2n = 10x = 80 [46, 61].

Although natural interspecific hybridization is uncommon in *Trifolium*, there have been several studies showing that it is possible [15, 56, 57, 103]. The ISH breeding programmes within the genus commenced over 50 years ago. Two common objectives from these breeding programmes were to understand the evolutionary relationships within the genus and to introgress desirable traits into the species [3]. *Trifolium occidentale* is a diploid (2n = 16), perennial stoloniferous species that is closely related to white clover (*Trifolium repens*) [3, 103]. It is indigenous to the coastal areas of Portugal, Spain, France and the British Isles, hence its tolerance to saline and dry habitats. This trait provides a potential source of drought-tolerant genes that could be used to improve white clover [45]. The ISH of *Trifolium* species crossed with white clover will allow elite germplasm to be bred with alleles that are not present in white clover populations [3]. Pederson and McLaughlin [74] performed a variety of crosses between *Trifolium* species, and *T. occidentale* x *T. repens* hybrids were the only fertile hybrids, which also showed resistance to peanut stunt virus. However, often the two species do not cross easily and result in near-sterile triploid hybrids [45]. Using 4x *T. occidentale* has yielded more successful crosses, resulting in significant advances in the introgression of drought and salt tolerance traits to white clover [21, 37, 74, 101].

Pedigrees are used in plant breeding to visualise the breeding crosses and the transmission of alleles responsible for trait expression and consequent breeding patterns [86]. They are a crucial first step in identifying genetic bottlenecks in breeding populations, and integration with genomics has increased their relevance in plant breeding programmes even further [5]. The significance of plant pedigrees is increasing as the need to lift the rate of genetic gain in white clover [43], increase the range of its environmental adaptation and increase its tolerance to plant stressors. Pedigrees are also used alongside molecular studies to increase the accuracy of molecular phylogenetic studies [23]. Pedigree maps can also be used to identify germplasm variation which can be utilised in future prebreeding decisions [29, 30]. For this to occur effectively, knowledge of population structure and relatedness coefficients are the first steps [49].

Prebreeding is becoming an increasingly important prerequisite of plant breeding programmes. Plant species that focus on new variation and flow of allelic variation benefit from prebreeding research. Related species that with desirable traits in the genus *Trifolium* are often used in prebreeding efforts and have been increasingly utilised in recent years. We used historical pedigree data from the *T. ambiguum, T. arvense, T. dubium, T. hybridum, T. medium, T. subterraneum* and the *T. repens* x *T. occidentale* ISH collections held at the Margot Forde Germplasm Centre (MFGC) in Palmerston North, New Zealand. The objectives of this study were (i) to create a pedigree map for each *Trifolium* species (hereafter referred to as *Trifolium spp.*), (ii) to analyse patterns affecting inbreeding and kinship, and (ii) to investigate variation in the collection that can be potentially utilised in future prebreeding work.

## Results

### Pedigree map sizes and complexity

Between 0 and 3 generations were traced among *Trifolium* spp., with *T. subterraneum* and *T. repens* x *T. occidentale* having most generations. The least number of generations (0–1) was observed in *T. arvense* and *T. medium* (Table 1). The shallowness of the pedigree maps is due to the low number of generations and reflects low breeding activity. The completeness of parentage was variable (Table 2) with *T. repens* x *T. occidentale* having the greatest number of accessions with complete parentage (39.3%). *T. subterraneum* had the greatest number of accessions with null parentage (47.6%). Terminal lines are described as accessions that are not involved in any further breeding and reflect on the amount of breeding activity. With the highest number of terminal lines in the pedigree (765 accessions, 94% of total) belonging to *T. ambiguum*, and the smallest number ((235 accessions, 50% of total), being in *T. subterraneum*. The breeding activity peaked in the 1990’s due to an influx of accessions being deposited at the MFGC. This is from both breeding crosses and accessions introduced from international collection trips. Visual inspection of the pedigree maps did not suggest any bottlenecks (data not shown).

**Table 1:** Number of generations, offspring distribution and number of terminal and orphan lines for seven *Trifolium* species at the Margot Forde Germplasm Centre, New Zealand

Species	Number of generations	Offspring distribution	Number of accessions with offspring	Non zero average offspring range	Non zero average offspring	Number of orphan lines	Number of terminal lines	Year of entry of the first accession
*T. ambiguum*	0–2	0–122	6	2–122	39	42 (5.2%)	765 (94.0%)	1962
*T. arvense*	0–1	0–5	4	1–5	2	32 (36.4%)	52 (59.1%)	1962
*T. dubium*	0–2	0–25	31	1–25	1	24 (11.2%)	160 (74.8%)	1956
*T. hybridum*	0–2	0–7	27	1–7	1	66 (18.1%)	270 (74.2)	1955
*T. medium*	0–1	0–39	14	1–13	5	5 (4.5%)	93 (83.0%)	1939
*T. subterraneum*	0–3	0–3	13	1–2	1	225 (47.6%)	235 (49.7%)	1956
*T. repens* x *T. occidentale*	0–3	0–65	75	1–32	6	732 (49.7%)	1054 (71.6%)	2015

**Table 2:** Completeness of parentage information of germplasm of seven *Trifolium* species from the Margot Forde Germplasm Centre, New Zealand. Half parentage indicates that one parent is listed

Species	Number of accessions in pedigree map	Number of accessions used in parameter analysis (% from total)	Full parentage (% from total)	Half parentage (% from total)	Null parentage (% from total)
*T. ambiguum*	814	772 (94.8%)	0 (0.00%)	772 (94.8%)	42 (5.2%)
*T. arvense*	88	55 (62.5%)	0 (0.00%)	56 (63.6%)	32 (36.4%)
*T. dubium*	214	191 (89.3%)	1 (0.5%)	190 (88.8%)	23 (10.8%)
*T. hybridum*	364	196 (76.6%)	1 (0.3%)	297 (81.6%)	66 (18.1%)
*T. medium*	112	107 (95.5%)	29 (25.9%)	78 (69.6%)	5 (4.5%)
*T. subterraneum*	473	248 (52.4%)	0 (0.00%)	248 (52.4%)	225 (47.6%)
*T. repens* x *T. occidentale*	1472	643 (43.7%)	579 (39.3%)	594 (40.4%)	299 (20.3%)

The smallest range of offspring distribution (0–3) was in *T. subterraneum*, compared to the largest distribution of *T. ambiguum* (0–122) (Table 1). Within accessions that had offspring, the highest average number of offspring (39) belonged to *T. ambiguum*, indicating a high level of breeding activity. This contrasts to *T. subterraneum, T. hybridum* and *T. dubium* which had an average offspring number of 1, showing low breeding activity.

Accessions with large numbers of offspring families were also identified. Only one accession of *T. ambiguum*, ‘AZ2640’ (*k* = 0.025), contributed 34 half-sib and full-sib offspring families to the population. This accession had the half parentage of ‘AZ1981’, which was an introduction from New South Wales, Australia in 1988 and is known as cultivar ‘Monaro’.

A total of four accessions of *T. dubium*, AZ1840, AZ170, AZ2022, and AZ1649, contributed 93 progeny (44%) to the population. Accession AZ1840 was an introduction into the MFGC in 1984 from the Manawatu region of New Zealand and produced 20 half-sib and full-sib offspring families. Accessions AZ170, AZ2022 and AZ1649 contributed 22, 22 and 29 half-sib and full-sib offspring families respectively. Accession AZ2022 was an introduction from the Manawatu region, and accession AZ1649 was an introduction from Portugal in 1983.

Two accessions, AZH1605 and C25897, contributed a large number of half-sib and full-sib offspring families to the *T. repens* x *T. occidentale* ISH, population. Accession AZH1605 (k = 0.008) was known as a BC1F2 hybrid with accession AZH784 as its parent. Accession C25897 was bred in 2010 with accession C25638 as its parent. The white clover cultivars, ‘Grasslands® Mainstay’ and ‘Grasslands® Kopu II’, were listed among the lineage. Mainstay white clover is a large-leaved, high yielding white clover that has high dry matter yield [7]. Kopu II is a large-leaved, high yielding white clover which has a high tolerance to clover root weevil [104]. as the aim of the ISH was to increase yield, tolerance to pests and drought tolerance, it is sensible that these two cultivars were among the parents.

### Influencing accessions and introductions

Relevant parents are described in this study as accessions that have structured large portions of the pedigree. *T. ambiguum, T. dubium, T. hybridum* and *T. medium* had two relevant parents compared with *T. arvense* and *T. repens* x *T. occidentale* ISH which had three relevant parents and *T. subterraneum* which had four relevant parents.

#### *T. ambiguum*

**Fig. 1:**
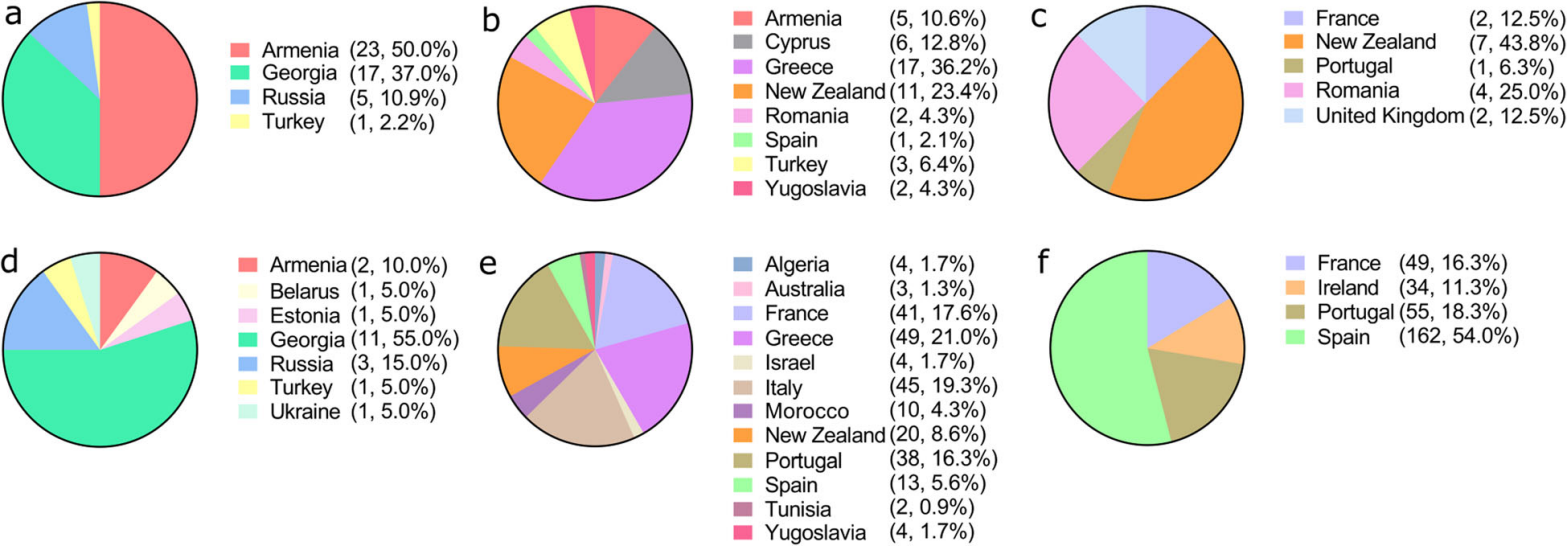
Total number of introductions into the Margot Forde Germplasm Centre germplasm collection from listed geographic locations for *T. ambiguum* (**a**), *T. arvense* (**b**), *T. dubium* (**c**), *T. hybridum* (**d**), *T. subterraneum* (**e**) and *T. repens* x *T. occidentale* ISH(f)

Two relevant parents, AZ1981 and AZ104, from *T. ambiguum* influenced the population structure of the species. Accession AZ1981 was introduced in 1985 from New South Wales, Australia. It is better known as cultivar Monaro. Accession AZ104 was also an introduction from Australia in 1962 from the organisation Commonwealth Scientific and Industrial Research Organisation. The introductions from *T. ambiguum* were from Armenia, Georgia, Russia and Turkey (Fig. 1a).

#### *T. arvense*

A total of three relevant parents, AZ1353, AZ2855 and AZ2925 were discovered for *T. arvense*. Accession AZ1353 was introduced into the MFGC in 1979. Accession AZ2855 was introduced in 1989 from the Yugoslavian Forage Legume Collection. Accession AZ2925 was an introduction from Hawkes Bay in 1990 and was collected from verges and cliffs. Hawkes Bay is a region in the east coast of New Zealand’s North Island that generally has a warm and relatively dry climate. The northern and central bays of the region include hilly coastal land. The species is often present in areas with sandy soil, sand dunes, and in areas that are not irrigated. The influence of the founding accession AZ2925 could be attributed to the strong adaptation of the accession to an environment similar to its native range. Country of origin was recorded for 47 introductions for *T. arvense* (Fig. 1b).

#### *T. dubium*

A total of two relevant parents, AZ753 and AZ2022, from *T. dubium*. Accession AZ753 was an introduction from Portugal in 1975. Accession AZ2022 was introduced into the MFGC in 1986 and was from a pasture selection from the Manawatu region in New Zealand. The summer climate of Manawatu is temperate, as is the semi-arid climate of Portugal. *T. dubium* had 16 introductions with a recorded country of origin (Fig. 1c).

#### *T. hybridum*

A total of two relevant parents, AB75 and AB230 were identified for *T. hybridum*. Accession AB75 was a Mackenzie country, New Zealand collection and was introduced into the MFGC in 1973. Accession AB230 was collected from Belarus in 1975. Both Belarus and the Mackenzie country have very distinct seasons; long dry summers and cold snowy winters. *T. hybridum* had 20 introductions with a recorded country of origin (Fig. 1d).

#### *T. medium*

A total of two relevant parents, Z1 and Z127, were identified for *T. medium*. Accession Z1 was an introduction from the former USSR in 1939. Z127 was introduced into the MFGC in 1985 and was categorised as a breeding line.

#### *T. subterraneum*

A total of four relevant parents, AK1213, AK452, AK799 and AK334, were identified for *T. subterraneum*. Accession AK334 was introduced into the MFGC in 1956 and was an early flowering type. Accession AK452 was introduced into the MFGC in 1962 and was a collection from Morocco. Accession AK799 was a collection from France in 1987 and was subject to a flowering/formononetin breeding selection. Accession AK1213 was collected from South Australia in 1993.

*T. subterraneum* had the largest number and accessions from different countries. The prominent countries of origin from the highest to lowest number of accessions were Greece (49), France (41), Portugal (38) and New Zealand (20), Spain (13) and Morocco (10) (Fig. 1e).

#### *T. repens* x *T. occidentale* ISH

The ISH, *T. repens* x *T. occidentale,* had three relevant parents, AZH776, AZH784 and AZH761. Accession AZH776 was introduced into the MFGC and had the commercial cultivar ‘Durana’ in the parentage [14]. Accessions AZH784 and AZH761 entered the MFGC in 2016. The parentage for both accessions are two commercial cultivars, ‘Kopu II’ and ‘Durana’. Kopu II is a large-leaved white clover cultivar that is high yielding and recovers rapidly after grazing [94]. Durana is a small-leaved white clover cultivar that originated from the United States [14]. A total of 300 parental accessions had the country of origin information. The largest number of introductions were made in *T. occidentale* x *T. repens* with 162 parental accessions from Spain and 55 from Portugal (Fig. 1f).

### Diversity and inbreeding

**Fig. 2:**
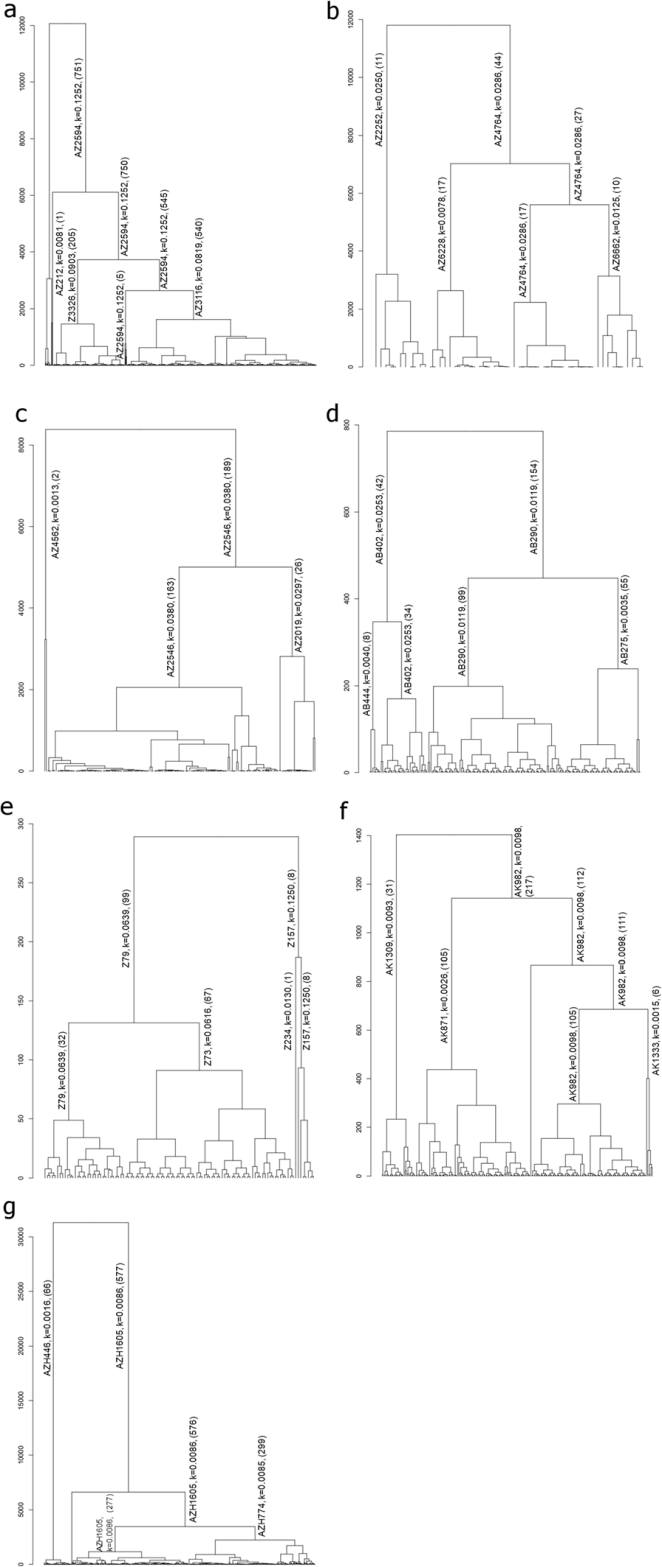
Dendrograms drawn based on a distance matrix of seven *Trifolium* species; *T. ambiguum* (**a**), *T. arvense* (**b**), *T. dubium* (**c**), *T. hybridum* (**d**), *T. medium* (**e**)*, T. subterraneum* (**f**) and *T. repens* x *T. occidentale* interspecific hybrids (**g**). Accessions indicated on the fusion points are influential parents determined by highest average kinship (**k**) in the clade. The number of accessions within clades are indicated in parentheses

The number of accessions that influenced the population structure for the *Trifolium* species varied (Fig. 2a-g). Three accessions influenced *T. dubium* (AZ4562 (*k* = 0.001), AZ2546 (*k* = 0.038) and AZ2019 (*k* = 0.029)) and *T. repens* x *T. occidentale* ISH (AZH446 (*k* = 0.001), AZH1605 (*k* = 0.008) and AZH1884 (*k* = 0.008)), whilst four accessions influenced *T. ambiguum (*AZ212 (*k* = 0.008), AZ2594 (*k* = 0.125), AZ3326 (*k* = 0.09) and AZ3116 (*k* = 0.081))*, T. arvense (*AZ2252 (*k* = 0.025), AZ6228 (*k* = 0.007), AZ4764 (*k* = 0.028) and AZ6662 (*k* = 0.012))*, T. hybridum (*AB444 (*k* = 0.004), AB402 (*k* = 0.025), AB290 (*k* = 0.011) and AB275 (*k* = 0.0035))*, T. medium (*Z79 (*k* = 0.063), Z73 (*k* = 0.061), Z234 (*k* = 0.012) and Z157 (*k* = 0.125)) and *T. subterraneum (*AK871 (*k* = 0.002), AK982 (*k* = 0.009), AK1309 (*k* = 0.009) and AK1333 (*k* = 0.001))*.*

Clustering patterns in the dendrograms could be explained by country of origin in some species. In *T. arvense,* cluster 1 had Turkey (11), cluster 4 had Turkey (1), New Zealand (5) and Romania (1), whilst Cluster 3 had Cyprus (3), Armenia (3), Yugoslavia (1) and Greece (9) (Fig. 2b). In *T. dubium,* cluster 4 had three countries with one accession from, New Zealand, France and Portugal. Cluster 3 had Romania (2), New Zealand (2), France (1) and the UK (1) (Fig. 2c). *T. subterraneum* had the most detailed geographic information associated with accessions. Cluster 2 had 64 accessions that weree associated with a specific country; Morocco (5), New Zealand (7) Algeria (2), France (1), Greece (22), Israel (2), Italy (4), Portugal (12), Spain (6), Tunisia (1) and Yugoslavia (2). In comparison, cluster 4 had 15 accessions associated with a listed country; France (1), New Zealand (2), Italy (4) and Portugal (8) (Fig. 2f).

The presence of commercial cultivars in the lineage of influencing accessions could be reasoning behind the divergence of population structure. Accession AZ3116 in *T. ambiguum* had the half parentage of AZ2640. It was known as the ‘Monaro late flowering selection’. Monaro is a high performing Australian cultivar, which has however, poor seed yield. However, breeding has progressed using hexaploid Monaro lines to improve the seed yield [96]. Comparatively, in *T. repens x T. occidentale* ISH, accession AZH446 had the half parentage listed of C25897 and a commercial white clover cultivar, ‘Trophy’. Accession AZH1884 had the full parentage listed of AZH1608/AZH1605. The accession AZH1605 had the ancestor of Kopu II and Durana, and AZH1884 had the ancestor Durana. Both Kopu II and Durana are commonly used commercial varieties in the New Zealand pastoral sector.

#### Half kinships, indirect relationships and unrelated accessions

Overall mean kinship in the *Trifolium* species did not exceed 2.3%; *T. ambiguum* (0.009), *T. arvense* (0.004), *T. dubium* (0.005), *T. hybridum* (0.001), *T. medium* (0.022), *T. subterraneum* (0.0002) and *T. repens* x *T. occidentale* (0.004). Unrelated accessions (*k =* 0), indirect relationships and half kinships (*k =* 0.5) were identified from pairwise calculations. *T. hybridum* (99.68%), *T. subterraneum* (99.15%) and *T. repens* x *T. occidentale* ISH (99.56%) had the highest number of accessions with *k =* 0, compared with *T. ambiguum* (97.12%)*, T. arvense* (95.26%), *T. dubium* (97.02%) and *T. medium* (91.03%). Whereas, *T. ambiguum* (0.74%), *T. arvense* (0.65%)*, T. dubium* (0.59%) and *T. medium* (5.75%) had the highest number of accessions with *k =* 0.25, contrasted with *T. hybridum* (0.09%)*, T. subterraneum* (0.003%) and *T. repens* x *T. occidentale* (0.07). Half kinships (*k =* 0.5) were found at the highest level in *T. arvense* (0.52%) and *T. medium* (1.37%) in comparison to *T. ambiguum* (0.04%)*, T. dubium* (0.27%)*, T. hybridum* (0.19%)*, T. subterraneum* (0.05%)*, T. repens* x *T. occidentale* (0.06%).

## Discussion

### *T. ambiguum* pedigree map complexity

The lineage of the cultivar Monaro had a large contribution to the pedigree of *T. ambiguum*. Monaro is a hexaploid (6x = 48) Australian cultivar described as vigorous and productive. It is suitable for most environments, especially areas where there is periodic summer drought [35, 73, 92]. Virgona and Dear [92] examined the performance of Monaro 11 years after establishment in south-eastern Australia against *T. repens* and *T. subterraneum*. Monaro was superior in legume content, digestibility and ability to respond to additional fertiliser.

The large root and rhizome systems of *T. ambiguum* and its well described tolerance to drought ([73, 92], Sheaffer and Seguin, 2009 [87]) has prompted its crossing with white clover (*T. repens* x *T. ambiguum*). White clover has a shallow root system which leads to underperformance in drought conditions. The *T. repens* x *T. ambiguum* ISH breeding programme has shown success in integrating larger root systems into the hybrid progeny [2, 58]. Abberton et al. [2] showed that BC3 hybrids were outperforming the other hybrids, *T. ambiguum* and *T. repens* in root characteristics. Marshall et al. [59] showed that comparisons of above-and-below ground biomass for BC1 and BC2 hybrid plots showed more dry matter in roots and rhizome of clover than in the *T. repens* plot.

### Influencing accessions and prebreeding traits

Over the period (1950–2016) there have been many international introductions of *Trifolium* species into the MFGC (Fig. 1). The introduction of germplasm from other countries has increased the variation in the MFGC’s clover collection and is crucial for maintaining the improvement of elite clover material. Introduced germplasm is important in prebreeding programmes and often become the parents that influence the population structure.

*T. subterraneum* is one of Australia’s most important forage legumes and has adapted to the harsh Mediterranean climates in specific parts of Australia [66, 67]. Introductions from the semi-arid conditions of South Australia and Morocco can provide insight into the adaptation extremes of subterranean clover. Having the adaptation to dry climates can be beneficial for drought-tolerant breeding programmes. In New Zealand, this is a prominent expectation of subterranean clover and is being trialled on dry areas of both hill and high-country farms to replace the use of white clover where irrigation is not available [53–55, 95]. Using more drought-tolerant *T. subterraneum* will increase its versatility in location of sowing and ability to withstand and persist in dry conditions.

The *T. repens* x *T. occidentale* ISH had introductions from countries that have dry, arid conditions, suitable for drought-tolerant phenotypes. The common objective of breeding programmes for *T. subterraneum* and *T. repens* x *T. occidentale* is to introgress drought tolerance traits, often through breeding for persistence in the former and larger root systems in the latter.

Kopu II was present in the pedigree of the *T. repens* x *T. occidentale* ISH. Kopu II is a large leaf, highly persistent and yielding white clover that has rapid recovery after grazing and elevated sugar levels (Ref). A crucial advantage of Kopu II is the high tolerance to clover root weevil, which is becoming an increasing problem in pastoral systems [104]. Durana was bred specifically to be highly persistent under grazing pressure and to tolerate acidic soils [14]. The commonality of the two commercial cultivars having persistence as the key objective could be what is influencing the ancestors to be at the top of the clusters. While white clover has suitable persistence for multiple pastoral systems, *T. occidentale* is not as broadly adapted. By using the cultivars that are very high in persistence in an ISH, it could reduce the risk of losing the persistence traits in recombination events.

Climatic and geographical similarities between the influencing accessions in all *Trifolium* spp. is strong reasoning behind the divergence and influence of the founding accessions. The regions and countries associated with accessions provide insight into desirable phenotypic advantages. There were several accessions originating from Australia, which has several climate zones due to the latitudinal span of the country, ranging from temperate to tropical climates. However, the largest area of Australia is desert and semi-arid. Therefore, the phenotypes of plant introductions from Australia are often drought tolerant and adapted to arid climates. Accessions originating from the Mediterranean region will have similar adaptations to Australia due to similar climates [13]. The Mediterranean has dry summers and rainy winters, often producing collected ecotype accessions with drought-tolerant phenotypes.

There were three regions in New Zealand where accessions have originated from: Manawatu, Hawkes Bay and Mackenzie country. The Manawatu region in New Zealand is not exposed to extreme climatic conditions. However, drought conditions can occur in summer [18]. The Hawkes Bay region is prone to rainfall and temperature variations. The summer season is drought-prone with prevailing winds [91]. The Mackenzie country is one of the driest areas of New Zealand. The region has a temperate-continental climate with long, hot summers and snow in winter [72]. Within the *Trifolium* spp. introductions, drought-tolerant traits are often a desirable adaptation to have and are a common breeding objective. As the species are used in pastoral systems in New Zealand, both irrigated and non-irrigated, it is often desirable that the species have summer drought tolerance.

### Diversity of *Trifolium* accessions

**Fig. 3:**
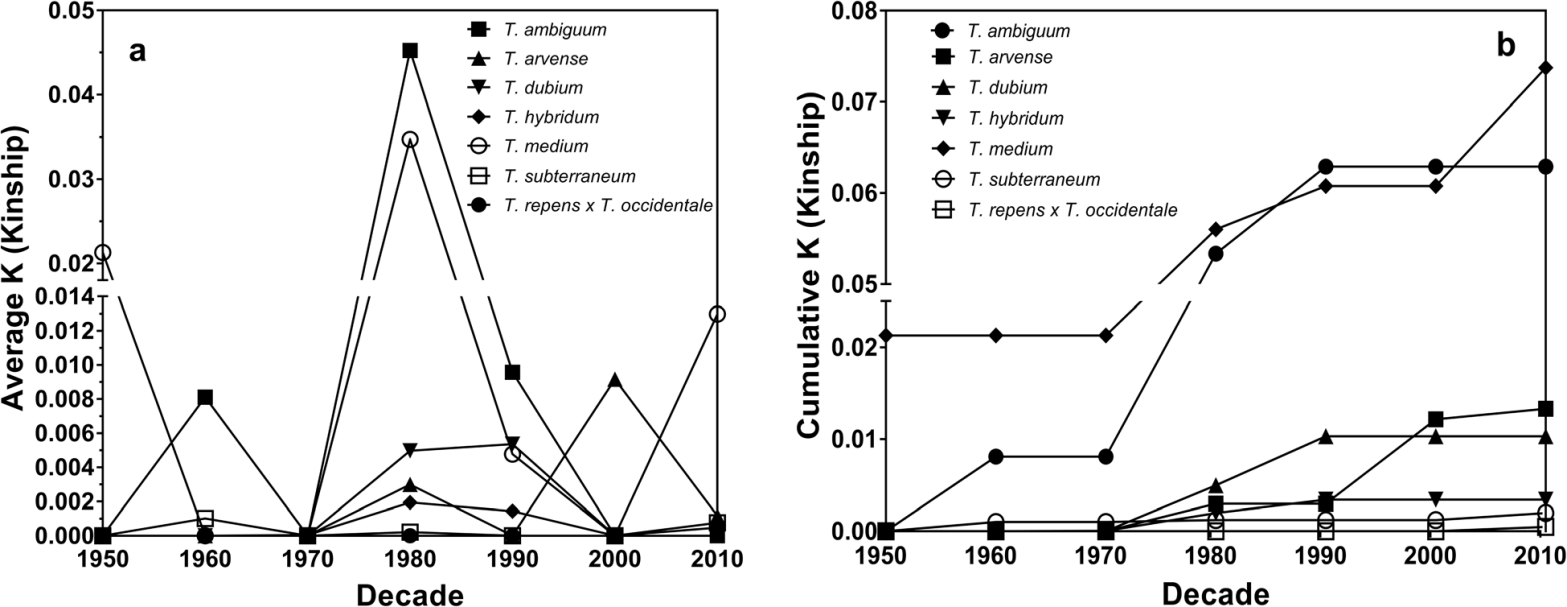
The trend in average (**a**) and cumulative (**b**) kinship in seven *Trifolium* species across seven decades

The kinship values indicate that genetic relatedness within *Trifolium* spp. is low (Fig. 3). However, kinship, as derived in this study, overestimates genetic diversity because we assume that all relevant parents are unrelated, as they are giving rise to different clusters in the population structure. However, this does not have to be true. Crucial genes that control species-specific characteristics (e.g., leaf size and trifoliate leaf) must be homologous so that the individual can be classified as a member of the species [39]. Kinship may overestimate genetic diversity because of unaccounted relationships due to the lack of bookkeeping when recording crosses. In all *Trifolium* spp. reported here, there were accessions with null parentage listed (Table 2). These were excluded from the genetic parameter analysis due to the questionable results of whether a zero kinship was from null parentage or a true zero k value. The correlation of estimates of genetic diversity based on pedigree data have been reported for other plant species [51, 64]. However, missing data in the parental information can influence correlations to be poor, with higher correlations being expected when the available pedigree information is more detailed [51]. El-Kassaby et al. [31] argues that when looking at a pedigree on a species level, not all crosses need to be accurately recorded as the pedigree is producing a large overview of population-specific patterns, rather than within cluster or accession analysis. If the purpose of the study is to analyse within cluster or accession patterns, it is crucial that the pedigree data is complete or near-complete.

Interestingly, a large proportion of accessions in *Trifolium* spp. had an average kinship coefficient of 0, showing that they are completely unrelated to any accession within the species. This indicates useful crosses that could be planned across breeding pools to generate new variation. Populations with a wider genetic base and greater adaptation could produce offspring with important agronomic traits. Within species diversity is a topic of high importance to many pastoral research groups because of the documented benefits of greater genetic variation [80].

Intrapopulation genetic diversity is important for the long-term persistence of breeding programmes for two main reasons: 1) phenotypic variation is correlated with adaptive potential of populations, and 2) neutral genetic variation of natural populations reflects inbreeding and genetic drift, which reduces the variation within populations [12, 47, 76].

### Genetic resources for *Trifolium* improvement

The *Trifolium* family is held in many genebanks globally, comprising of landraces, accessions, breeding lines, wild relatives and commercial cultivars [1, 62, 77, 78]. However, it has been noted that often *Trifolium* collections are biased towards the two major species, red and white clover. The first clover entry into the MFGC was in 1931 and 1897 in the United States collection. In the USDA collection red and white clover contribute to 56% of the collection. Wild relatives are present; however, the presence is poor for species that are possible gene sources. Only 4.5% of the collection is species that are close relatives to red and white clover [62].

The results from the *Trifolium* species in this study are comparable to the results by [29, 30], where diversity within the white and red clover accessions at the MFGC were assessed. Overall, the level of variation was high across all of the *Trifolium* species accessions. These results are encouraging as the mating systems of *Trifolium* species are outcrossing and self-incompatible. Utilising the available variation within the accessions will assist breeders in developing populations for specific breeding targets. While it is useful to assess variation within each species, it is optimal to compare the results across species to harness all available variation for prebreeding and hybridisation programmes.

The high levels of diversity found in the genebank collections reported in white [29] and red clover [30] are positive for future hybridisation programmes. Prebreeding efforts in minor *Trifolium* species could complement white and red clover breeding [29]. showed that the country of origin of accessions has influence on the population structure. Hybridisation programmes of major *Trifolium* species with characterised germplasm of minor species could utilise that information and increase the efficiency of crosses. The now-defined breeding pools in this study could be exploited for between- and-within pool crosses with white and red clover.

### Prebreeding from related species for *Trifolium* improvement

Prebreeding is an essential bridge between genebanks and breeding programs. Prebreeding is defined as all activities designed to identify useful characteristics from unadapted germplasm. The goals of prebreeding can be broadly classified as widening the base of diversity and to increase plant production through various traits. The programs developed from prebreeding can generate new base populations with the end goal of cultivar development. When used in hybrid programs, heterotic patterns can be identified.

The most prominent example of prebreeding success in *Trifolium* is the development of the white clover ISH [101]. Williams et al. [102] identified the 10 *Trifolium* species that could be used in hybridisation with white clover. It was thought that these species could bring useful traits to white clover as the minor species often have useful genes that can contribute to increased genetic gain [3, 100]. Hoyos-Villegas et al. [43] investigated the rate of genetic gain in white clover in a selection of 80 white clover cultivars released between 1920 and 2010 across 17 countries. Their study showed that there has been less than 0.17% gain per decade in both yield and content. The three species that have been popularly bred as hybrids are *Trifolium uniflorum*, *Trifolium occidentale* and *Trifolium ambiguum*. *Trifolium uniflorum* have been successful in breeding programmes and have shown promising results in producing hybrids that have increased performance under drought tolerance and reduced phosphate levels [44, 68–71].

There has been less research performed for *Trifolium pratense* ISH, however, *Trifolium medium* has been popular for integrating perenniality into red clover [3, 61, 81, 82]. Isobe et al. [46] created four backcross generations of *T. pratense* x *T. medium* ISH. The BC_4_ plants had a 61% survival rate by the fourth year of the field trial and almost all BC_4_ plants produced mature seed. Female fertility was 21.3% and pollen fertility was 65.3%. These results showed the potential for these hybrids to be used in future red clover selection programs.

### Challenges in prebreeding

The ability for the germplasm held in the genebanks to respond to differing abiotic and biotic stresses is well recognised. However, as promising as prebreeding is, there are big challenges too. The two biggest challenges are time and reducing linkage drag. A large time commitment is needed to identify useful germplasm and hybridise it with well-adapted germplasm whilst reducing the unwanted genes [88]. The time commitment is also largely dependent on the information on the accessions at the beginning of the prebreeding programme [63]. The ability to determine whether germplasm is compatible is also time consuming, especially if there is lack of knowledge around wild species. Linkage drag is a major limiting factor in prebreeding and Sharma et al. [85] states that it is the most important factor responsible for low use of germplasm. Linkage drag is defined as the unplanned inheritance of undesirable genes along with the target genes due to their close linkage [5]. Generating large populations and utilising genomic tools can help to overcome linkage drag [9, 84, 85].

## Conclusions

We found that the genetic resources held in the *Trifolium* spp. at the MFGC have a wide genetic base. The pedigree maps constructed, and derived relatedness parameters estimated showed that seven *Trifolium* spp. in the MFGC have high diversity within the recorded germplasm. The absence of inbreeding in all species highlights the available genetic diversity and is a positive insight into what forage breeding has achieved. There were no visual bottlenecks in the pedigree maps.

Germplasm exchange between countries and domestic and international collection trips has proved successful in creating a collection of accessions of *Trifolium* spp. for the MFGC. Influencing ancestors have been identified and relevant parents that influenced population structure have been distinguished. The results from interrogating the pedigrees showed that geographical origin seemed to be influencing the international introductions. Knowing the country of origin of accessions that contributed large numbers of progeny and resulted in elite material, and obtaining the breeding pools to which they belong, can provide knowledge on diverging phenotypic characteristics. This will enhance future prebreeding decisions [106]. Introductions from countries with semi-arid environments strongly influenced the population structures and adaptation to target environments drove divergence between the clusters of accessions.

Overall, the results from this research on the population structure of *Trifolium* spp. are relevant to pre-breeding efforts. Although *Trifolium* spp. are not as largely used as *T. repens* and *T. pratense* in agricultural systems, climate change and societal demands for sustainable agriculture will require that new traits are integrated into *T. repens* and *T. pratense* via interspecific hybridization. Species such as *T. ambiguum* can hybridise with white clover, and *T. medium* with red clover, to improve production and survival, particularly in marginal environments. The *T. repens* x *T. occidentale* ISH has been successful in the improvement of white clover root systems. With a known population structure, the future pre-breeding decisions can be more efficient and defined breeding pools will maximize germplasm utilisation. Breeders can make use of the assembled pedigree maps reported here to strategize how to maximize genetic variation and incorporate pre-breeding efforts in breeding programs. Despite the challenges associated with prebreeding, it is critical that time and funding is invested into prebreeding programmes. Utilising wild relatives will strengthen breeding programmes and accelerate the rate of genetic gain and the release of cultivars to market.

## Methods

### Germplasm

The *Trifolium* spp. that were selected for this study were selected based on historical breeding activity and their importance in New Zealand’s pastoral systems. The formal identification of the germplasm used in this study was undertaken by the MFGC. The germplasm is available in the MFGC. A total of six species: *T. arvense, T. subterraneum, T. ambiguum, T. dubium, T. hybridum* and *T. medium* have been the subject of active breeding programmes since the 1950’s. The *T. repens* x *T. occidentale* ISH breeding programme has pedigree information available from 2015. International collection trips to collect germplasm from abroad in these species have been successful in bringing new germplasm back to the MFGC. Where necessary, permits were obtained for collections. Whilst these species are currently not major pastoral species used in New Zealand, some are often used in pastoral mixes or have been critical in research for improving the major species *T. repens*. One species, *T. ambiguum*, and one hybrid, *T. repens* x *T. occidentale*, have been actively used in the *Trifolium* ISH breeding programme and have improved root systems in *T. repens*. These species were chosen as (i) they are often used in pastoral systems as minor pastoral species or, (ii) they are used thoroughly in pastoral research as a research species or, (iii) they can be hybridised with a major pastoral species.

### Data filtering

Pedigree map construction was undertaken using pedigree data from the MFGC database. Seven minor *Trifolium* spp. were used in the pedigree analysis, chosen based on the size and completeness of the data available (Table 2). The methodology used in this study is the same as in Egan et al. [29] and Egan et al. [30]. In short, accessions have been introduced over a range of decades, from 1950 to 2010. A range of breeding techniques have been used during population development over this period, including bi-parental cross and polycross methods. The pedigree maps were constructed using Helium, a software that allows the visualisation of large pedigrees [86]. Accessions with large numbers of offspring families were identified. These largely contributing accessions were determined as parents with several progeny well-above the mean number of progenies for each species. The term “accession” here is used to refer to any seed material entered in the MFGC as a distinct population with an identification number.

A smaller number of accessions were used in the derivation of population parameters than the number used to construct the pedigree map, because of the completeness of pedigree information available. The accessions were categorised based on identifiable parental information, full or half parentage indicated, and the type of cross that was conducted (e.g., biparental crosses). Polycrosses represented a small number of accessions and were excluded from the parameter subset. This was due to the software package not having an option for handling the pedigree data with both biparental crosses and polycrosses. Further, polycrosses do not fit the allele frequency expectations of biparental crosses and could therefore skew estimates of relatedness as indicated in Egan et al. [29] and Egan et al. [30].

### Data analysis

The R package ‘pedigree’ was used to calculate the number of offspring, kinship and inbreeding [22].

The pedigree information was used to calculate kinship (*k*) by using a recursive application of two formulae:

1 $$ {F}_{yy}=\frac{\left(1+F{m}_y{f}_y\right)}{2} $$

2 $$ {F}_{xy}=\frac{\left({F}_{xmy}+{F}_{xfy}\right)}{2} $$

where *F* is the coefficient of inbreeding, *F*_*yy*_ is the kinship of *y* with *y* and *F*_*xy*_ is the kinship of two individuals, assuming *x* is not a progeny of *y*. When *x* and *y* are founding accessions, F_xy_ = 0. The random inheritance of one gene from each parent is transcribed as *my* and *fy* of *y*, and the relationship of these genes is *Fm*_*y*_*f*_*y*_ [33]. *k* is the level of relatedness of the accession. Heatmaps were used to visualise the pairwise combinations of relatedness in each *Trifolium* spp. (Supplemental material).

A dendrogram was used to visualise and summarise the clustering of the populations based on pedigree information and kinship coefficients. The influencing ancestors were termed by identifying the accessions with the highest mean kinship per cluster. This was verified by pedigree lineages.

Inbreeding was calculated using the formulae [24, 98, 105]:

$$ {F}_y=\frac{H_o-H}{H_o} $$

where *H* is the unconditional probability that *y* is heterozygous at any given locus. *H*_0_ is the conditional probability that *y* is heterozygous at a given locus, when the genes are not identical-by-descent [33].

## Supplementary information

**Additional file 1 **: **Supplemental Figure 1.** Title of data: Kinship heatmap of the seven *Trifolium* species at the Margot Forde Germplasm Centre; *T. ambiguum* (a), *T. arvense* (b), *T. dubium* (c), *T. hybridum* (d), *T. medium* (e), *T. subterraneum* (f) and *T. repens* x *T. occidentale* interspecific hybrids (g).

## Data Availability

Data and pedigree map queries are available upon request to the authors. Requests for MFGC germplasm may be considered depending on availability of germplasm and the purpose of the request.

## Abbreviations

MFGC: Margot Forde Germplasm Centre
ISH: Interspecific Hybrids
GM: Genetic Modification
BC1F2: First Backcross Second Generation
