# Supplementary figures and images for: Pedigree analysis of pre-breeding efforts in Trifolium spp. germplasm in New Zealand
Source: BMC Genet. 2020 Sep 14;21:104. doi: 10.1186/s12863-020-00912-9 (PMC7489199; doi:10.1186/s12863-020-00912-9)

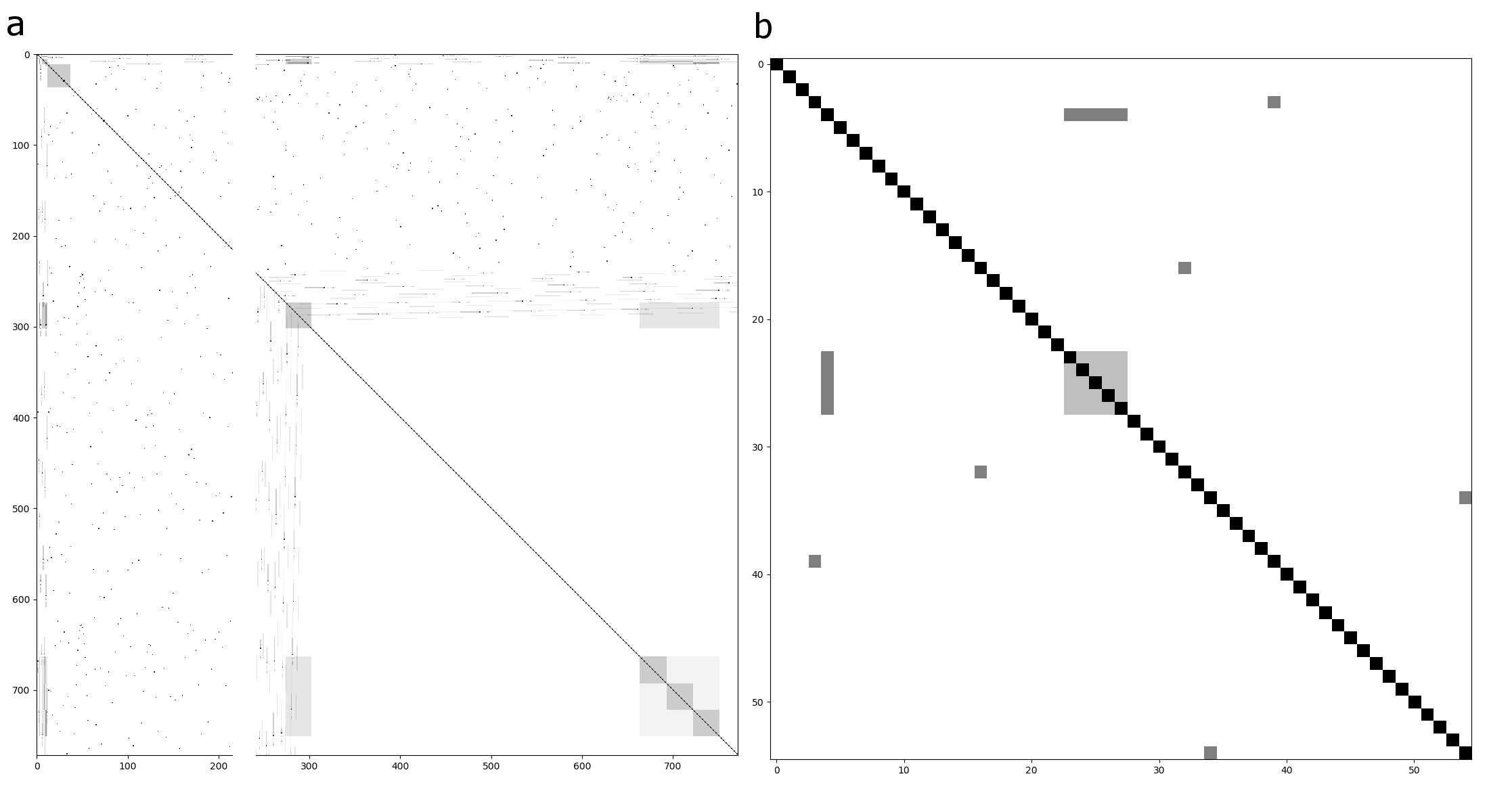

Supplement: Supplementary file 1 — Additional file 1 : Supplemental Figure 1. Title of data: Kinship heatmap of the seven Trifolium species at the Margot Forde Germplasm Centre; T. ambiguum (a), T. arvense (b), T. dubium (c), T. hybridum (d), T. medium (e), T. subterraneum (f) and T. repens x T. occidentale interspecific hybrids (g). [file 12863_2020_912_MOESM1_ESM.zip › Supplementary a and b.tif]

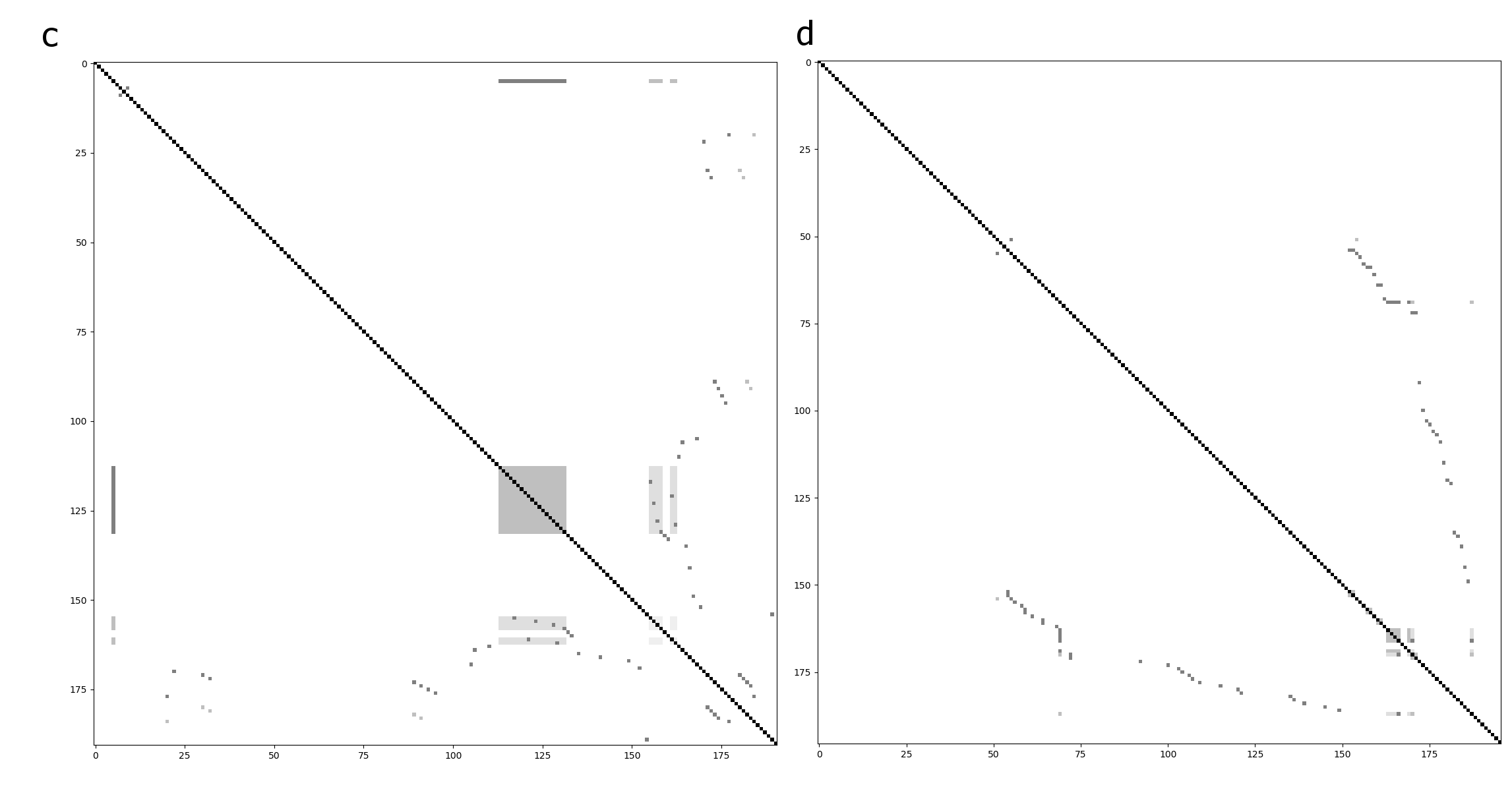

Supplement: Supplementary file 1 — Additional file 1 : Supplemental Figure 1. Title of data: Kinship heatmap of the seven Trifolium species at the Margot Forde Germplasm Centre; T. ambiguum (a), T. arvense (b), T. dubium (c), T. hybridum (d), T. medium (e), T. subterraneum (f) and T. repens x T. occidentale interspecific hybrids (g). [file 12863_2020_912_MOESM1_ESM.zip › Supplementary c and d.tif]

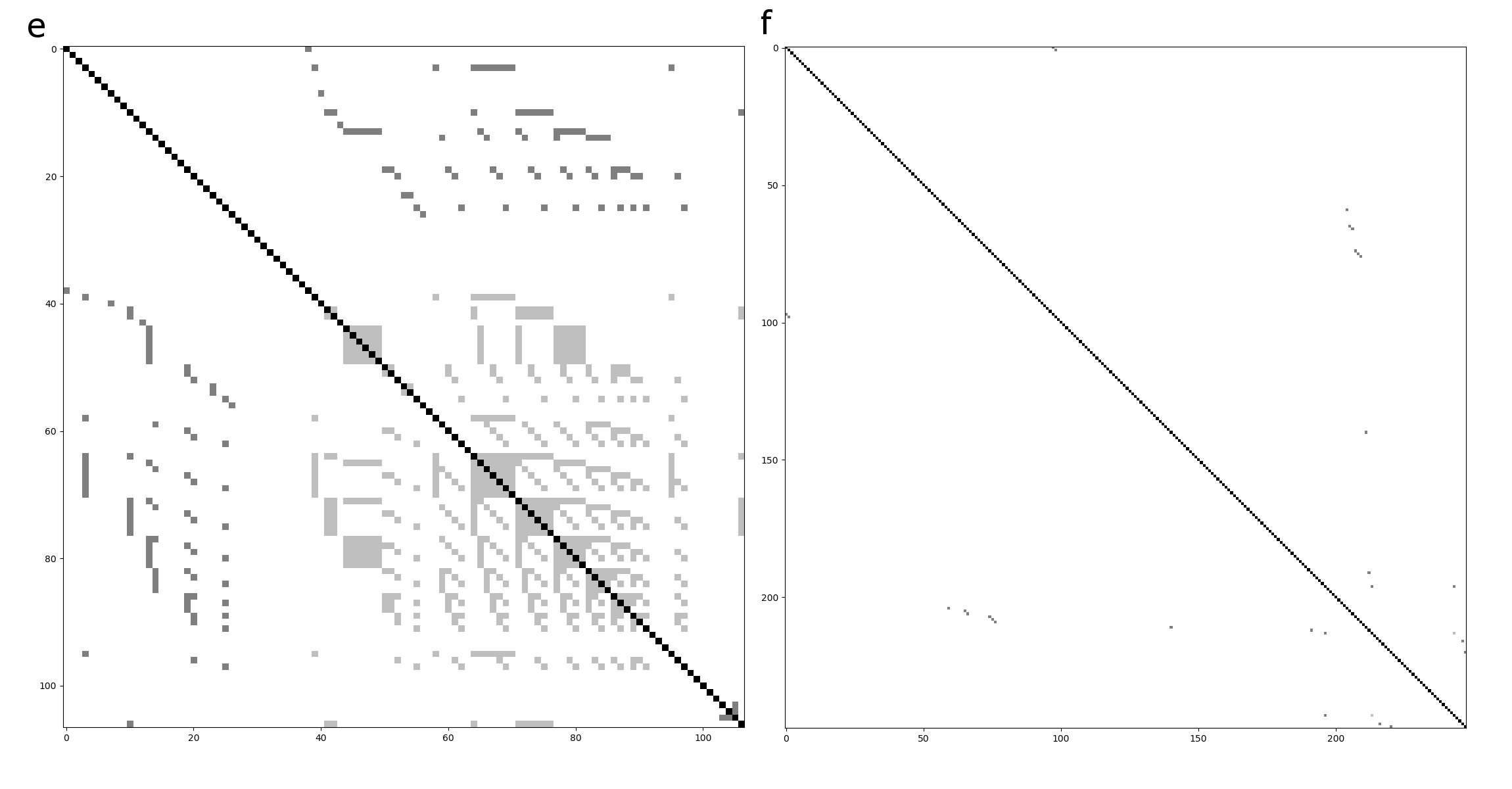

Supplement: Supplementary file 1 — Additional file 1 : Supplemental Figure 1. Title of data: Kinship heatmap of the seven Trifolium species at the Margot Forde Germplasm Centre; T. ambiguum (a), T. arvense (b), T. dubium (c), T. hybridum (d), T. medium (e), T. subterraneum (f) and T. repens x T. occidentale interspecific hybrids (g). [file 12863_2020_912_MOESM1_ESM.zip › Supplementary e and f.tif]

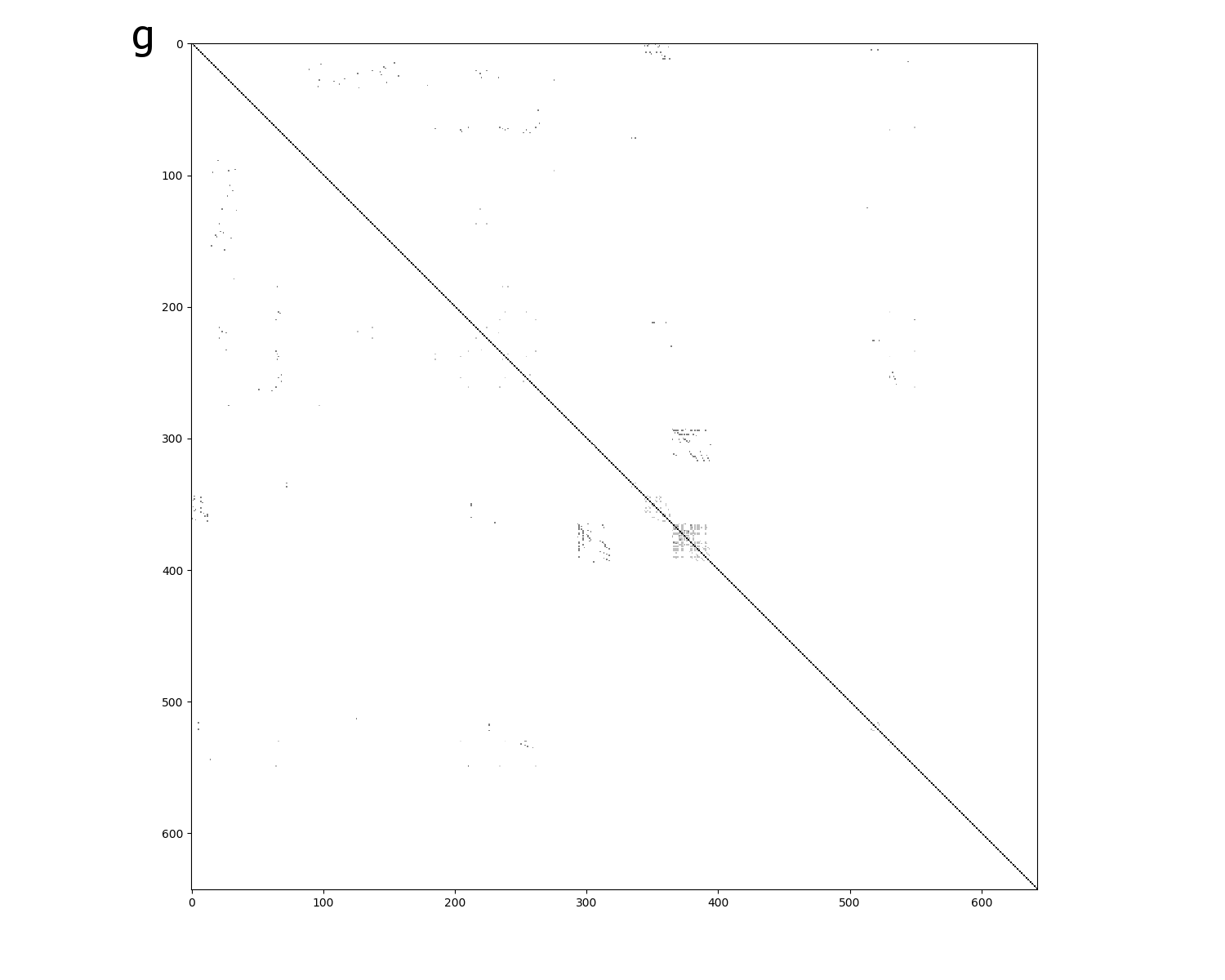

Supplement: Supplementary file 1 — Additional file 1 : Supplemental Figure 1. Title of data: Kinship heatmap of the seven Trifolium species at the Margot Forde Germplasm Centre; T. ambiguum (a), T. arvense (b), T. dubium (c), T. hybridum (d), T. medium (e), T. subterraneum (f) and T. repens x T. occidentale interspecific hybrids (g). [file 12863_2020_912_MOESM1_ESM.zip › Supplementary g.tif]
